# Regulatory Coherence and Professional Competency Development in Nursing: A Scoping Review and Policy Analysis of the Mexican Health System in an International Context

**DOI:** 10.3390/healthcare14142091

**Published:** 2026-07-13

**Authors:** María Esther Rodríguez López, Mercedes Gómez del Pulgar, Máximo González-Jurado, Fabiana Maribel Zepeda Arias, Yamile Anaya-Jiménez, Alina Renghea, Miguel Ángel Cuevas Budhart, Alfonso Meneses Monroy

**Affiliations:** 1 Facultad de Enfermería, Fisioterapia y Podología, Universidad Complutense de Madrid, 28040 Madrid, Spain; esther_rguez_@hotmail.com (M.E.R.L.); maximo.gonzalezjurado@gmail.com (M.G.-J.); ameneses@ucm.es (A.M.M.); 2 Department of Nursing, Faculty of Health Sciences, Universidad Francisco de Vitoria, 28223 Madrid, Spain; m.gomezdelpulgar@ufv.es (M.G.d.P.); a.renguea@ufv.es (A.R.); 3 Coordinación de Enfermería, Instituto Mexicano del Seguro Social, Mexico City 06600, Mexico; maribel.zepeda@imss.gob.mx; 4 Facultad de Estudios Superiores Iztacala, Universidad Nacional Autónoma de México, Tlalnepantla 54090, Mexico; anaya.yamile@gmail.com; 5 Unidad de Investigación Médica en Enfermedades Nefrológicas, Hospital de Especialidades, Centro Médico Nacional Siglo XXI, Instituto Mexicano del Seguro Social (IMSS), Mexico City 06720, Mexico

**Keywords:** nursing regulation, professional competencies, scope of practice, health policy, regulatory frameworks, advanced nursing practice, health systems, workforce deployment

## Abstract

**Background:** Nursing regulation is a key component of health system governance; however, its role in structuring the development and deployment of professional competencies remains insufficiently examined, particularly in emerging health systems. Objective: To map and synthesize the evidence on nursing regulation and its relationship with professional competencies across the nursing profession, including the development of advanced nursing roles, and to analyze the Mexican regulatory framework within an international context. **Methods:** A scoping review was conducted using a structured and transparent approach. Searches in Scopus, Web of Science, and PubMed identified 194 records. After duplicate removal and screening, 17 studies met inclusion criteria based on relevance to regulation, scope of practice, and competency development. In parallel, 13 regulatory instruments in Mexico were identified through targeted searches of official sources; 9 key documents were selected based on specificity and relevance. Data were analyzed using a policy-oriented framework. **Results:** Regulatory frameworks consistently define scope of practice and enable the translation of competency-based education into recognized clinical roles. Regulatory coherence was associated with expanded professional autonomy, advanced role development, and improved workforce integration. Fragmented regulatory environments were linked to misalignment between education and practice and underutilization of competencies. In Mexico, a broad but fragmented regulatory architecture was identified, lacking a unified regulatory axis and an autonomous professional body. **Conclusions:** Nursing regulation functions as a structural determinant of professional competency deployment. Strengthening regulatory coherence is essential to align education, practice, and health system needs, with implications for workforce performance, patient safety, and system sustainability.

## 1. Introduction

The regulation of professional nursing practice constitutes a core component of health system governance, as it defines the scope of practice, professional responsibilities, and mechanisms for public protection. Beyond its legal function, regulation operates as a key health policy instrument shaping the quality, safety, and efficiency of care, while enabling the effective translation of professional competencies into clinical practice [1,2].

Health systems have undergone profound transformations driven by population aging, the increasing burden of chronic diseases, and growing care complexity. In this context, the scope of nursing practice has expanded, encompassing both the development of advanced nursing roles and the growing influence of master’s and doctoral level education in shaping the content and boundaries of clinical practice; however, evidence indicates that such expansion is sustainable only when supported by coherent regulatory frameworks that clearly define roles, responsibilities, and accountability mechanisms [3,4].

Comparative evidence shows that regulation is a key determinant in the implementation of professional competencies. Countries with explicit regulatory frameworks demonstrate greater integration of nursing into care delivery and recognition of advanced roles, whereas fragmented or underdeveloped regulation generates gaps between competency-based education and actual clinical practice [5,6].

For the purposes of this review, advanced nursing roles are understood as nursing functions that extend beyond generalist, entry-level practice through expanded clinical decision-making, prescriptive, or care-coordination authority, generally acquired through graduate-level education; advanced nursing practice designates the broader professional category encompassing these roles, as conceptualized internationally by the International Council of Nurses [7]. In Mexico, this category has not yet been formally codified as a distinct professional designation within the national regulatory framework. Postgraduate nursing programs, including hospital specializations and master’s degrees, address advanced clinical and managerial competencies but do not consistently incorporate explicit content on the legal and regulatory framework that governs the scope of advanced practice, a curricular gap that contributes to the broader disconnect between competency-based education and its formal recognition in clinical settings.

Despite this evidence, important gaps remain. Existing literature has largely focused on high-income settings and specific regulatory reforms, with limited attention to how fragmented regulatory systems influence competency deployment. Moreover, regulation has predominantly been examined as a legal or administrative function rather than as a structural determinant of health system performance (that is, a foundational, system-level condition that shapes how professional competencies are authorized, distributed, and deployed across the health workforce, thereby influencing how effectively the health system as a whole functions).

In Mexico, nursing has undergone significant professionalization through the expansion of higher education and competency-based training. However, this evolution has not been accompanied by a unified regulatory framework. Instead, regulation is dispersed across multiple legal and technical instruments (that is, formal legal, professional, and institutional mechanisms, including legislation, licensure procedures, official technical standards, accreditation requirements, and professional certification guidelines that together govern nursing education and practice), without a specific professional law or an autonomous regulatory body, potentially limiting the effective deployment of competencies in practice [8].

In Mexico, regulatory authority over nursing is distributed across several governmental and professional institutions rather than concentrated in a single nursing-specific regulatory body. Professional licensure (cédula profesional) is granted by the Secretaría de Educación Pública through the Dirección General de Profesiones, certifying that an individual has completed an accredited nursing program, whereas the technical and clinical standards that govern the actual practice of nursing are issued separately by the Secretaría de Salud, primarily through Normas Oficiales Mexicanas such as NOM-019-SSA3-2013 [9,10,11]. Continuing certification and recertification of professional competencies, in turn, are overseen by professional certifying bodies under voluntary rather than statutory recognition. This separation between the authority that licenses the profession, the authority that defines clinical standards, and the bodies that certify ongoing competence reflects a broader pattern observed across the Region of the Americas, where statutory nursing councils with autonomous authority over licensure, discipline, and scope of practice remain the exception rather than the rule [8]. The absence of a single regulatory axis in Mexico is therefore not an isolated gap but part of a wider regional configuration in which nursing regulation has historically developed within general health and education legislation rather than as a profession-specific statutory framework.

Given the heterogeneity of available evidence, a scoping review is appropriate to systematically map scientific literature, policy analyses, and regulatory documents, providing a comprehensive overview of the extent and nature of existing knowledge [12,13].

This study is further guided by a policy analysis framework based on Walt and Gilson [14], which examines health policy through the interaction among context, content, process, and actors, enabling a structured interpretation of regulatory dynamics within health systems.

Therefore, the objective of this scoping review is to map and synthesize the available evidence on nursing regulation in Mexico and its relationship with the development and deployment of professional competencies, identifying key gaps and implications for health policy and practice.

## 2. Materials and Methods

### 2.1. Study Design

A scoping review was conducted to systematically map the available evidence on nursing regulation and its relationship with professional competencies, with a specific focus on the Mexican context. This methodological approach is appropriate for addressing complex and heterogeneous bodies of literature, including scientific studies, policy analyses, and regulatory documents, allowing for the identification of key concepts, knowledge gaps, and research priorities [8,12].

The review was conducted in accordance with the Preferred Reporting Items for Systematic Reviews and Meta-Analyses extension for Scoping Reviews (PRISMA-ScR) guidelines (see Supplementary Table S1) [13].

### 2.2. Eligibility Criteria

Sources were considered eligible for inclusion if they addressed nursing regulation, scope of practice, professional competencies, advanced nursing roles, or health policy. The review included peer-reviewed scientific articles, policy analyses, and official regulatory documents at both national and international levels. Mexican legal and regulatory instruments, as well as international institutional frameworks and policy reports, were incorporated to ensure a comprehensive analysis of regulatory structures and their implications for professional competency development. Mexican legal and regulatory instruments, as defined above, were established a priori as a distinct eligible source category alongside scientific literature, rather than identified incidentally during the search process. Eligible sources were required to be published between January 2000 and December 2025 and to be available in English, Spanish, or Portuguese. This 25-year window was applied to scientific articles, policy analyses, and gray literature in order to capture contemporary developments in nursing regulation and competency-based education relevant to the present health policy context while excluding outdated empirical evidence. Foundational Mexican legal instruments that remain currently in force, including the 1917 Constitution (art. 4 and 5) and the 1945 Regulatory Law of Article 5, were included independently of this temporal window and of their original enactment date, since they constitute the binding constitutional and legal basis that still governs professional practice today; these instruments were evaluated in their current, most recently reformed text rather than their original historical version.

Sources were excluded if they consisted of editorials, opinion pieces, or commentaries lacking analytical content, if they did not present clear authorship or institutional endorsement, or if they were not directly related to the regulation of professional nursing practice or the development and recognition of professional competencies.

### 2.3. Sources of Information

The literature search was conducted using multiple sources to ensure comprehensive coverage of both scientific evidence and regulatory frameworks.

Scientific literature databases. The search was performed in three major international bibliographic databases: PubMed, Scopus, and Web of Science. These databases were selected to ensure broad and high-quality coverage of peer-reviewed scientific literature related to nursing regulation, professional competencies, scope of practice, and health policy.

Gray literature sources. Targeted searches were conducted using Google Scholar to identify relevant gray literature, including policy analyses, institutional reports, and regulatory discussions not indexed in traditional biomedical databases. This approach enabled the inclusion of complementary evidence particularly relevant to health policy and professional regulation.

National regulatory documents. Official Mexican legal and regulatory instruments were retrieved from authoritative government sources, including the Diario Oficial de la Federación (dof.gob.mx) and the official repositories of the Secretaría de Salud and the Secretaría de Educación Pública. These documents were selected based on their legal validity and direct relevance to the regulation of professional nursing practice.

International institutional frameworks. Documents issued by international organizations, such as the World Health Organization and the International Council of Nurses, were included to provide a comparative and global perspective on nursing regulation and competency development, identified through the official institutional repositories and publications portals of the World Health Organization (https://www.who.int/) and the International Council of Nurses (https://icn.ch/).

In addition to scientific literature, the review incorporated relevant normative and policy documents to capture the regulatory dimension of nursing practice. These included official Mexican legal and regulatory instruments, as well as international institutional frameworks, which were selected based on their relevance and contribution to understanding the relationship between regulation and professional competencies.

### 2.4. Search Strategy

The search strategy was designed to identify literature addressing nursing regulation, professional competencies, scope of practice, and health policy, with particular attention to the Mexican context and its international comparators. A combination of free-text terms was used, structured around three core conceptual domains: regulation, competencies, and health systems.

Searches were conducted between October and December 2025, with the final search performed on 15 December 2025. The search strategy was adapted to each database according to its indexing system and search functionalities. Boolean operators (AND, OR) were used to combine terms and optimize the balance between sensitivity and specificity.

Given the policy-oriented nature of the review, no restrictions were applied regarding study design. The search process was iterative, allowing for refinement of terms based on preliminary results to improve relevance.

In parallel, complementary searches were conducted to identify normative and regulatory documents. These included manual searches of official repositories such as the Diario Oficial de la Federación, as well as institutional and international sources, in order to capture legal frameworks and policy documents not indexed in bibliographic databases.

The complete search strategies for each database are provided in Table 1 to ensure transparency and reproducibility.

### 2.5. Selection of Sources

The selection of sources followed a structured but flexible process consistent with the exploratory nature of the scoping review. All records identified through database searches were compiled and organized for screening. Duplicate records were identified and removed prior to analysis.

The selection process was conducted in two stages. First, titles and abstracts were reviewed to assess their relevance to the study objectives. Second, full-text documents were evaluated to confirm their inclusion based on their contribution to understanding nursing regulation, professional competencies, scope of practice, or health policy. Screening at both stages was performed manually by two reviewers working independently, using a structured spreadsheet matrix to record inclusion and exclusion decisions for each record, rather than a dedicated systematic-review software platform such as Covidence; discrepancies were resolved through discussion until a consensus was reached.

In addition to scientific literature, normative and regulatory documents identified through manual searches were incorporated into the selection process. These documents were evaluated based on their institutional validity, relevance to the Mexican regulatory context, and their contribution to the analytical framework of the study.

Given the policy-oriented nature of the review, the study selection prioritized conceptual relevance and analytical contribution rather than strict methodological hierarchies.

### 2.6. Data Charting

Data extraction was conducted using a structured approach tailored to the heterogeneity of the included sources. The following information was collected: author and year of publication, country or region, type of source (scientific article, policy analysis, or regulatory document), thematic focus, and key findings related to regulation and professional competencies. For normative documents, additional information regarding legal scope and institutional origin was considered.

### 2.7. Data Synthesis

A qualitative thematic synthesis was conducted to identify patterns, relationships, and gaps across the included sources [15]. The analysis focused on the structure and evolution of regulatory frameworks, the recognition and implementation of professional competencies, and the relationship between regulation, education, and clinical practice.

Data coding and theme development followed a combined inductive-deductive process [16]. Each included source, whether a scientific study or a regulatory document, was first read in full and summarized in a structured matrix capturing study or document characteristics, key findings, and the explicit or implicit relationship described between regulation and professional competencies. Recurring concepts were inductively coded as descriptive labels, such as role recognition, fragmented authority, or competency-practice gap, and codes sharing a common conceptual focus were iteratively grouped into broader analytic themes through constant comparison across sources. These themes were then deductively mapped onto the four interacting dimensions of the Walt and Gilson policy analysis framework, namely context, content, process, and actors, in order to interpret how the regulatory features identified across sources relate to professional competency deployment. The resulting themes and their cross-cutting relationships were synthesized to develop an integrated conceptual model.

The synthesis was guided by a policy analysis framework based on Walt and Gilson [14], which conceptualizes health policy through the interaction of context, content, process, and actors. This approach enabled a structured interpretation of how regulatory systems influence the development and operationalization of professional competencies.

## 3. Results

**Figure 1:**
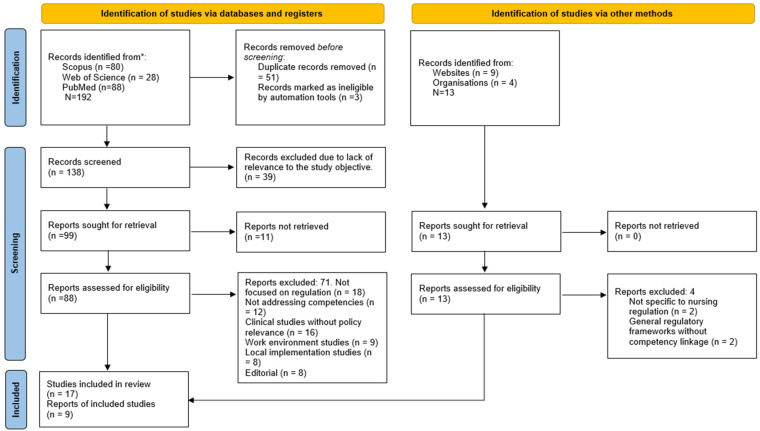
PRISMA-ScR flow diagram of the study selection process. Adapted from the PRISMA 2020 statement flow diagram template [17].

A total of 194 records were identified across Scopus (n = 80), Web of Science (n = 28), and PubMed (n = 88). After removing 51 duplicates, 143 records were screened. Following title, abstract, and full-text assessment, 17 studies met the inclusion criteria. In addition, 9 key regulatory and normative documents were incorporated through targeted searches of official sources. The detailed selection process is presented in the PRISMA-ScR flow diagram (Figure 1).

Excluded studies were primarily those not addressing nursing regulation or professional competencies, including editorial materials, studies focused on work environment variables, local implementation reports without a regulatory perspective, and clinically oriented studies not relevant to the study objective.

The included studies consisted mainly of policy analyses, comparative studies, and integrative or narrative reviews, with an international scope encompassing OECD countries, Europe, Latin America, Africa, and global health systems. Despite methodological heterogeneity, all studies addressed the relationship between regulatory frameworks, scope of practice, and competency development.

### 3.1. International Scientific Evidence on Nursing Regulation and Professional Competencies

The synthesis of the included studies shows a consistent pattern: nursing regulation functions as a structural determinant of the development and effective deployment of professional competencies within health systems.

The 17 included studies, mainly policy analyses, comparative studies, and integrative reviews conducted across diverse international contexts, consistently examined the relationship between regulatory frameworks, scope of practice, and competency development [3,18,19].

Across these settings, regulation defines the scope of practice and enables the translation of competency-based education into clinically recognized roles [2,20]. In contexts with coherent regulatory frameworks, greater integration of nursing into care delivery has been observed, particularly through expanded roles and task redistribution [4].

In contrast, fragmented or weak regulatory environments are associated with misalignment between education and practice, limiting the use of competencies and restricting professional autonomy [21,22]. This is especially evident in low- and middle-income countries, where insufficient regulatory articulation constrains role development [19,23].

At the system level, regulation operates as a governance mechanism shaping workforce capacity and health system performance [20,24]. Overall, the evidence indicates that regulation acts as a functional mechanism that conditions how competencies are recognized, authorized, and implemented, with regulatory coherence emerging as the key linking factor between education, professional roles, and clinical practice (Table 2).

### 3.2. Legal and Regulatory Framework of Nursing in Mexico

The Mexican regulatory framework for nursing is characterized by a multi-level structure integrating constitutional, legal, and technical instruments. At the constitutional level, Articles 4 and 5 establish the right to health and freedom of professional practice, providing the legal basis for professional accountability [31]. This foundation is operationalized through the General Health Law, which defines the role of health personnel within the National Health System [9], and its corresponding regulations governing service organization and delivery.

Regulatory development has been further strengthened through technical standards, particularly NOM-019-SSA3-2013, which defines roles, responsibilities, and core principles of nursing practice, including patient safety and ethical standards [11]. More recently, the General Law of Higher Education incorporated a competency-based approach, reinforcing the link between education and professional practice [10]. This competency-based approach applies broadly to nursing education at both undergraduate and graduate levels, rather than to a specific advanced practice category. At the postgraduate level, advanced nursing education in Mexico is mainly offered through hospital specializations (Especialidad en Enfermería) and master’s degrees (Maestría en Enfermería) in clinical, administrative, or educational areas; these programs strengthen advanced clinical and managerial competencies but are not currently linked to a formally regulated, distinct advanced practice nursing designation within the national legal framework.

Despite this broad regulatory framework, the analysis indicates a lack of structural coherence. Regulation is dispersed across general legislation, administrative provisions, and technical standards, without a specific professional law or an autonomous regulatory body for nursing. This fragmentation limits the clarity of scope of practice, constrains the recognition of advanced roles, and weakens the articulation between competency-based education and its implementation in clinical settings (Table 3).

The framework reflects a multi-level regulatory structure integrating constitutional provisions, general health legislation, technical standards, and educational policies. The analysis highlights the breadth of the regulatory system and its progressive incorporation of competency-based elements, as well as the absence of a unified regulatory body or specific professional law for nursing.

### 3.3. Thematic Synthesis and Development of the Integrated Conceptual Model

The thematic synthesis of the 17 international studies (Table 2) and the 9 Mexican regulatory instruments (Table 3) converged on a single cross-cutting theme: regulatory coherence functions as the structural condition that determines whether professional competencies, across the nursing profession broadly and not exclusively in advanced practice roles, are translated into recognized clinical authority. Three interrelated sub-themes emerged from this convergence. First, formalization of competencies, reflected in the analytical contributions of references [2,3,27], describes how explicit regulatory frameworks transform competencies acquired through education into legally recognized clinical authority. Second, workforce integration and task redistribution, drawn from references [4,18,20], describes how regulatory coherence enables the efficient allocation of professional roles within health systems. Third, fragmentation and underutilization, evident in references [19,21,22,29], captures how the absence of unified regulatory authority generates gaps between formal competencies and their clinical application, a pattern mirrored in the Mexican regulatory landscape described in Table 3, where constitutional, legal, and technical instruments coexist without a single governing axis.

**Figure 2:**
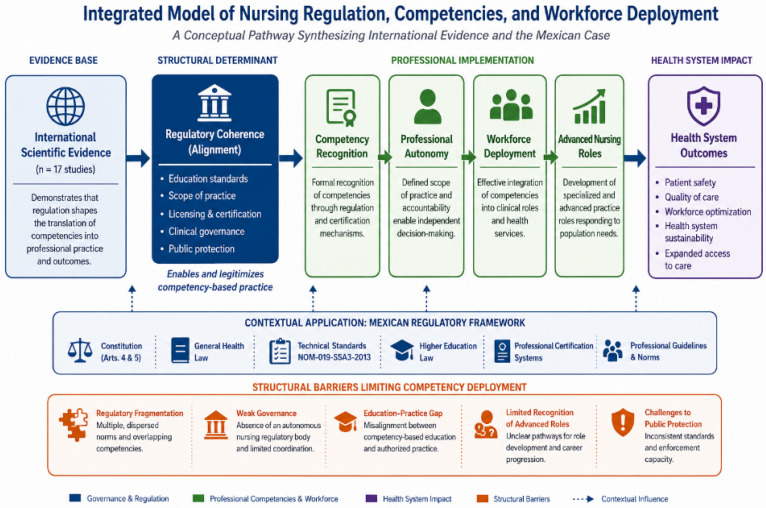
Integrated model of nursing regulation, professional competencies, and workforce deployment in nursing.

These three sub-themes were mapped onto the dimensions of the Walt and Gilson [14] policy analysis framework and used to construct the integrated conceptual model presented in Figure 2. Regulatory coherence was positioned as the central element of the model because it represents the point of convergence of the three sub-themes across both the international and the Mexican evidence base. The model’s input components correspond to the international evidence (n = 17 studies) and the Mexican regulatory instruments (n = 9 documents), while its output components, namely competency deployment, workforce integration, and health system performance, represent the consequences identified throughout the thematic synthesis when regulatory coherence is present versus absent. This synthesis process, summarized narratively here and operationalized in Table 2 and Table 3, is presented prior to the Discussion to make explicit how the empirical and regulatory evidence translates into the conceptual model that anchors the interpretation developed in Section 4.

Overall, the results indicate that nursing regulation, both in the international literature and in the Mexican context, functions as a structural determinant for the development, recognition, and effective deployment of professional competencies across the nursing profession as a whole, including but not limited to advanced practice roles. While Mexico has developed a broad and progressive regulatory framework, its main challenge lies in achieving greater coherence and integration across regulatory instruments to ensure that competency-based education is effectively translated into clinical practice.

Figure 2 presents an integrated conceptual model that synthesizes the international evidence (n = 17 studies) and the Mexican regulatory framework (n = 9 key documents). The model illustrates how regulatory coherence enables the translation of competencies into practice, whereas its absence leads to fragmentation, ambiguity in scope of practice, and limited recognition of advanced roles. In this context, regulatory coherence emerges as the central element linking education, professional roles, and health system performance.

The conceptual model was developed from the synthesis of 17 international studies and 9 key Mexican regulatory documents. The figure illustrates regulation as a structural determinant linking competency-based education with professional practice, highlighting the role of regulatory coherence in enabling effective deployment of competencies and identifying fragmentation as a key limitation in the Mexican context.

## 4. Discussion

This scoping review demonstrates that the regulation of professional nursing practice operates as a structural determinant of competency deployment within health systems, extending beyond its traditional interpretation as a legal or administrative framework. By integrating international evidence with the Mexican regulatory context, the findings show that regulation defines not only the formal scope of practice, but also the conditions under which competencies are legitimized, recognized, and effectively translated into clinical care [1,2].

From a policy analysis perspective, and consistent with the framework proposed by Walt and Gilson [14], regulation should be understood as a governance mechanism embedded within the interaction of context, actors, and institutional processes. This approach allows for the analysis to move beyond a purely descriptive narrative and to interpret, on the basis of the thematic synthesis presented in Section 3.3, how regulatory structures are associated with the configuration of professional roles, accountability mechanisms, and health system performance, rather than to establish a direct causal relationship.

International evidence consistently shows that competency-based education alone is insufficient without regulatory alignment. The expansion of nursing roles, particularly advanced practice, depends on explicit regulatory frameworks that formally recognize competencies and define their scope of practice [3,4]. Comparative analyses further demonstrate that regulatory coherence enables task shifting, improves workforce efficiency, and strengthens the integration of nursing into care delivery systems [6,18]. In contrast, fragmented regulatory environments generate persistent gaps between training and practice, leading to underutilization of competencies and limiting the contribution of nursing to overall system performance [8].

These regulatory gaps carry direct consequences for population health, both in Mexico and internationally. When competencies acquired through education cannot be fully exercised in practice, health systems forgo part of the workforce capacity in which they have already invested through training, which translates into reduced access to care, particularly in underserved or resource-constrained settings, and into missed opportunities for task shifting that could alleviate physician shortages and reduce waiting times for patients. In the Mexican context, where nursing constitutes the largest share of the health workforce serving a population exceeding 124 million people, this underutilization has a compounding effect on overall system capacity. Beyond its clinical consequences, this fragmentation also shapes the policy environment itself: when regulatory authority over nursing is dispersed and a profession-specific governing body is absent, policymakers may lack a single, accountable interlocutor able to articulate the scope, value, and developmental needs of the nursing workforce, which in turn limits their capacity to fully appreciate the complexity of health systems and the essential role nurses play in their effective functioning.

### 4.1. Regulatory Coherence as a Structural Condition for Competency Deployment

Within this context, the Mexican case offers a particularly relevant contribution to the international literature. Mexico has developed a broad and progressively constructed regulatory framework, integrating constitutional provisions, general laws, technical standards, and institutional guidelines. However, this regulatory architecture lacks structural coherence, as authority is distributed across multiple instruments without a unified governing axis or an autonomous regulatory body specific to nursing.

This results in a structural paradox in which regulatory abundance coexists with limited functional integration. Although multiple instruments define competencies and responsibilities, their lack of articulation generates ambiguity in scopes of practice, inconsistent recognition of competencies, and insufficient formalization of advanced nursing roles.

### 4.2. Implications for Clinical Practice and Health System Performance

These structural characteristics have direct implications for clinical practice. When competencies are not clearly defined and supported by regulatory frameworks, their implementation becomes dependent on local institutional interpretations rather than standardized policies. This variability affects the effective deployment of highly trained professionals, limits the consolidation of advanced nursing roles, and constrains the integration of nursing within multidisciplinary care models.

From a health systems perspective, the implications are equally significant. In a country such as Mexico, where healthcare is delivered to a population exceeding 124 million people, the effective use of nursing competencies represents a strategic component of system sustainability. The absence of regulatory coherence limits workforce optimization, constrains the implementation of task shifting, and reduces the efficiency of service delivery.

Importantly, this challenge is not unique to Mexico. Similar patterns have been documented in other emerging and middle-income countries, where advances in competency-based education have outpaced regulatory adaptation [8], including Eswatini [23], Kenya [29], and other Latin American and Caribbean health systems [22,24]. In this sense, the Mexican case should be understood as a representative model of regulatory transition in large-scale health systems.

### 4.3. Implications for Health Policy and Regulatory Governance

These findings underscore that strengthening nursing regulation is fundamentally a matter of health policy and government responsibility. Regulation must be conceived as a dynamic instrument within health system governance, requiring continuous alignment with educational developments and service needs.

Achieving this alignment implies improving coherence across existing regulatory instruments, formally recognizing advanced nursing roles, and strengthening institutional mechanisms that support regulatory implementation. Positioning regulation as a central element of workforce policy is essential to maximize the contribution of nursing to health system performance, particularly in contexts characterized by increasing demand and constrained resources.

From an international perspective, this study contributes by demonstrating that the key challenge is not the absence of regulation, but the lack of integration and strategic alignment within existing regulatory systems. This distinction is particularly relevant for countries with large public health systems, where fragmented regulatory structures may limit the effective deployment of competencies despite significant advances in education.

### 4.4. Policy Recommendations

Building on the synthesis and policy analysis presented above, five specific recommendations can be drawn to strengthen nursing regulation in Mexico and to inform comparable reforms in other health systems. First, consolidate the currently dispersed constitutional, legal, and technical instruments identified in Table 3 into a unified, profession-specific regulatory framework, ideally anchored by an autonomous nursing council with statutory authority over licensure, scope of practice, and professional discipline. Second, formally define and regulate advanced nursing practice as a distinct professional category, building on the existing postgraduate specialization and master’s degree programs described in Section 3.2, so that competencies acquired through graduate education translate into legally recognized clinical authority. Third, harmonize the clinical and ethical provisions of NOM-019-SSA3-2013 with the competency-based provisions introduced by the General Law of Higher Education [10,11], ensuring that curricular competencies are explicitly mirrored in the technical standards that govern clinical practice. Fourth, link the continuing certification and recertification guidelines of 2023 to statutory recognition mechanisms so that the maintenance of competencies results in formal scope-of-practice updates rather than remaining a voluntary, non-binding process. Fifth, create a national, publicly accessible registry mapping the relationship between nursing competencies, regulatory instruments, and clinical roles, to provide policymakers with a single, consolidated reference for monitoring regulatory coherence and informing future legislative reform; an approach illustrated here for Mexico that could be adapted by other large-scale health systems facing comparable regulatory fragmentation.

### 4.5. Limitations

This study has several limitations. Although the scoping review methodology allowed for a structured and transparent mapping of diverse sources, it was not designed to be exhaustive, which may introduce some selection bias. Additionally, the analysis primarily relied on formal regulatory documents, which might not fully reflect how these regulations are implemented across different clinical settings. Moreover, the lack of empirical outcome data limits the ability to directly measure the impact of regulatory frameworks on clinical performance.

Nonetheless, these limitations should be considered within the context and goals of the study. By combining scientific literature with normative and regulatory analysis and anchoring the interpretation in a policy analysis framework, this work provides a solid and well-informed understanding of regulation as a key factor within health systems.

Importantly, the findings from the Mexican context offer valuable insights for other large-scale, resource-constrained health systems. Rather than being a limitation, analyzing a country with a complex and extensive public health infrastructure contributes to the international discussion by demonstrating how regulatory consistency is essential for optimizing workforce capacity and enhancing health system performance.

## 5. Conclusions

This scoping review shows that the regulation of professional nursing practice is a key factor for effectively using competencies within health systems, as it defines the scope of practice and helps translate competency-based education into safe and accountable clinical care.

The findings suggest that the main issue is not the lack of regulation, but rather the inconsistency and misalignment between regulatory frameworks, education, and clinical practice. The Mexican example demonstrates how a broad regulatory structure can limit its effectiveness when this integration is weak.

These findings have clear global relevance. Improving regulatory coherence is a strategic policy lever that may contribute to optimizing workforce capacity, enhancing quality of care, and improving health system performance, especially in large and complex health systems.

## Figures and Tables

**Table 1:** Databases, search strategy, and Boolean operators.

Database	Search Strategy
Scopus	TITLE-ABS-KEY (nursing AND (regulation OR legislation OR “scope of practice”) AND (“professional competencies” OR “nursing competencies” OR “advanced practice nursing”) AND (“health policy” OR governance))
Web of Science	TS = (nursing AND (regulation OR legislation OR “scope of practice”) AND (“professional competencies” OR “nursing competencies” OR “advanced practice nursing”) AND (“health policy” OR governance))
PubMed	((“Nursing”[Mesh] OR nursing[Title/Abstract]) AND (regulation[Title/Abstract] OR legislation[Title/Abstract] OR “scope of practice”[Title/Abstract]) AND (competenc*[Title/Abstract] OR “advanced practice nursing”[Title/Abstract]) AND (“Health Policy”[Mesh] OR “health policy”[Title/Abstract] OR “health systems”[Title/Abstract])) AND (“2000/01/01”[Date—Publication]: “2025/12/15”[Date—Publication])

Searches were conducted independently in each database using controlled vocabulary terms and free-text keywords adapted to the indexing structure of each platform. English and Spanish were used to capture both international evidence and Mexican regulatory literature relevant to the review aims. Duplicate records were identified and removed using Mendeley reference management software and manual verification procedures. The final literature search was conducted on 15 December 2025.

**Table 2:** Evidence on nursing regulation and its relationship with professional competencies.

Author(s) and Year	Country/Context	Methodological Design	Key Findings	Contribution to Regulatory Model	Analytical Integration into the Regulatory Framework
Benton et al. [2]	International	Comparative analysis	Regulation legitimizes competencies and ensures public protection	Establishes regulation as a foundation for professional recognition	Positions regulation as the mechanism that links competency validation with public trust and accountability
Delamaire & Lafortune [3]	OECD countries	Policy analysis	Advanced roles depend on formal regulatory frameworks	Links regulation with expansion of competencies	Demonstrates that without regulatory formalization, advanced competencies cannot be operationalized in practice
Maier & Aiken [4]	39 countries	Cross-sectional comparative study	Task shifting is associated with regulatory reforms	Shows regulation as driver of workforce optimization	Identifies regulation as a determinant of task redistribution and efficiency in health systems
Maier et al. [5]	Europe and North America	Governance analysis	Alignment of education, regulation, and organization is essential	Positions regulation within system coherence	Highlights regulation as a central axis integrating training systems with healthcare delivery structures
Kroezen et al. [21]	Europe	Comparative regulatory study	Regulatory variability affects scope of practice	Demonstrates impact on competency recognition	Shows that fragmented regulation produces unequal deployment of competencies across systems
Rafferty et al. [20]	Global	Health policy analysis	Regulation strengthens health systems’ sustainability	Frames regulation as structural determinant	Conceptualizes regulation as a macro-level governance tool influencing workforce capacity
Luna et al. [22]	Latin America	Regional review	Gap between training and regulation persists	Identifies misalignment in emerging systems	Highlights structural gaps between education systems and regulatory recognition in LMICs
Heale & Rieck Buckley [25]	International	Comparative review	Regulatory models shape APN development	Defines typologies of regulatory frameworks	Provides comparative models that explain variation in competency deployment globally
Scanlon et al. [19]	LMICs	Integrative review	Weak regulation limits APN implementation	Highlights regulatory gaps in developing contexts	Demonstrates that regulatory weakness constrains role expansion in resource-limited systems
Oldenburger et al. [24]	Latin America and Caribbean	Policy analysis	APN implementation requires regulatory coordination	Emphasizes governance and policy alignment	Positions regulation as a prerequisite for scalable implementation in emerging regions
Poghosyan et al. [26]	USA	Qualitative policy study	Regulatory barriers limit practice autonomy	Identifies operational regulatory barriers	Provides micro-level evidence of how regulation restricts competency deployment
Ladd & Schober [27]	Global	Policy analysis	Prescribing authority reflects regulatory scope	Connects competencies with legal authorization	Demonstrates how regulation translates competencies into legally recognized actions
Dlamini et al. [23]	Eswatini	Implementation study	Role development depends on regulatory alignment	Shows regulation in emerging health systems	Illustrates how regulation enables adaptation of roles in low-resource settings
Officer et al. [28]	International	Policy analysis	Policy mechanisms enable role implementation	Links policy design with competency deployment	Identifies regulatory instruments as facilitators of workforce transformation
Ndirangu-Mugo et al. [29]	Kenya	Gap analysis	Scope of practice not aligned with regulation	Demonstrates regulatory lag	Highlights mismatch between formal competencies and regulatory frameworks
Mathews et al. [4]	USA	Policy analysis	Removing legal barriers expands practice	Shows regulatory reform effect	Demonstrates how regulatory change directly expands scope of competencies
Beal et al. [30]	USA	Longitudinal regulatory study	Regulatory changes expanded scope over time	Demonstrates temporal evolution of regulation	Provides longitudinal evidence of how regulation evolves with professional roles

The last two columns represent the authors’ analytical synthesis regarding the contribution of each study to the understanding of nursing regulation as a structural determinant of professional competencies. APN: Advanced Practice Nursing; LMICs: Low- and middle-income countries.

**Table 3:** Summarizes the main regulatory instruments and their relationship with professional competencies.

Year	Regulatory Instrument	Scope of Application	Relationship with Professional Skills
1917 (current reforms)	Political Constitution of the United Mexican States (art. 4th y 5th)	National	Right to health and freedom of profession; constitutional basis for responsible professional practice.
1984	General Health Law	National Health System	It defines the provision of health services and the responsibility of health personnel; it supports clinical and quality competencies.
1986	Regulations of the General Health Law regarding the Provision of Medical Care Services	Public and private services	It regulates the organization of services, interdisciplinary work, and clinical management.
1945	Regulatory Law of Article 5 of the Constitution (professions)	Professional practice	It establishes requirements for professional practice and professional certification; a basis for the legal recognition of competencies.
2000	Internal Regulations of the Ministry of Health (and amendments)	Federal public administration	Define regulatory and normative powers in health, including nursing.
2013	NOM-019-SSA3-2013, For nursing practice in the National Health System	Professional nursing practice	Define roles, levels of responsibility, and principles of clinical competence, ethics, and patient safety.
2018	Agreements and guidelines for patient quality and safety	National Health System	They strengthen skills in safety, continuous improvement, and patient-centered care.
2021	General Law of Higher Education	Higher education	It incorporates the competency-based training approach and social relevance in nursing.
2023	Guidelines for professional certification and recertification (professional bodies)	Professional development	They promote continuous updating and validation of professional skills.

## Data Availability

The original contributions presented in this study are included in the article. Further inquiries can be directed to the corresponding author.

## Supplementary Materials

The following supporting information can be downloaded at: https://www.mdpi.com/article/10.3390/healthcare14142091/s1, Table S1: Preferred Reporting Items for Systematic reviews and Meta-Analyses extension for Scoping Reviews (PRISMA-ScR) Checklist.

## Abbreviations

The following abbreviations are used in this manuscript:

APN	Advanced Practice Nursing
DOF	Diario Oficial de la Federación
ICN	International Council of Nurses
LMICs	Low- and middle-income countries
NOM	Norma Oficial Mexicana
OECD	Organisation for Economic Co-operation and Development
PRISMA-ScR	Preferred Reporting Items for Systematic Reviews and Meta-Analyses Extension for Scoping Reviews
WHO	World Health Organization
